# Supplementary material for: Heat production in a feeding matrix formed on carrion by communally breeding beetles
Source: Front Zool. 2021 Feb 2;18:5. doi: 10.1186/s12983-020-00385-7 (PMC7851950; doi:10.1186/s12983-020-00385-7)
Supplement: Supplementary file 1 — Additional file 1 (supplementary figures). [file 12983_2020_385_MOESM1_ESM.docx]

Supplementary figures for

“Heat production in a feeding matrix formed on carrion by communally breeding beetles”

by S. Matuszewski & A. Mądra-Bielewicz


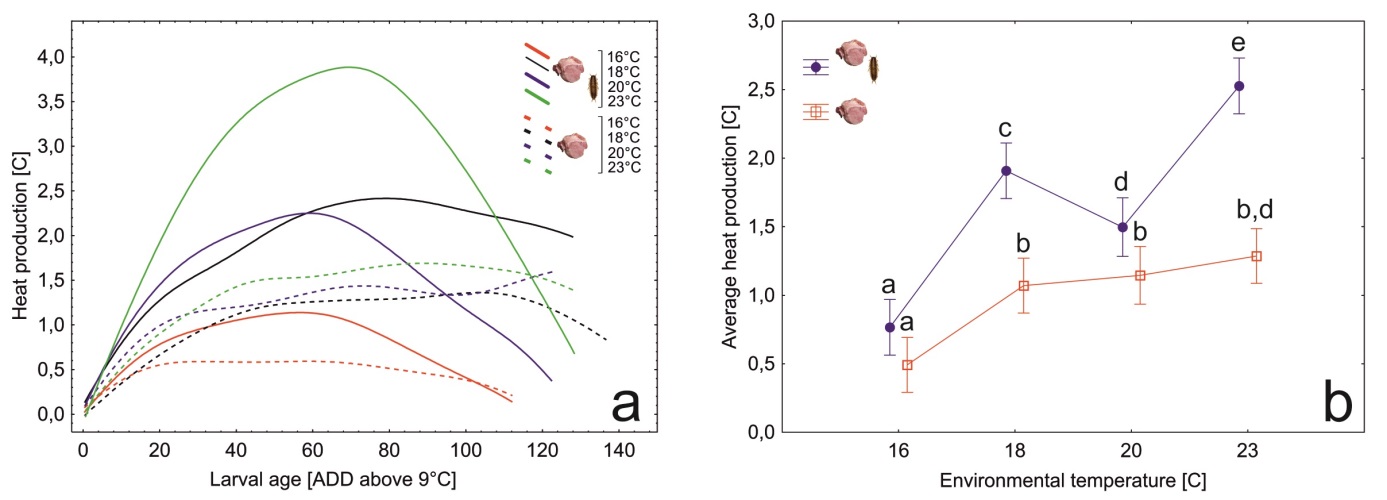


**Fig. I.** Longitudinal thermal profiles (**a**) and differences in the average heat production (**b**) of the feeding matrix between colonies of larval *Necrodes littoralis* reared under different constant environmental temperatures, compared against thermal profiles and heat production in meat decomposing without the beetles. Thermal profiles were fitted to the data using the distance-weighted least-squares smoothing procedure. ADD – accumulated degree-days, symbols – means, whiskers – 95% confidence intervals, different letters denote significant differences in pairwise comparisons.


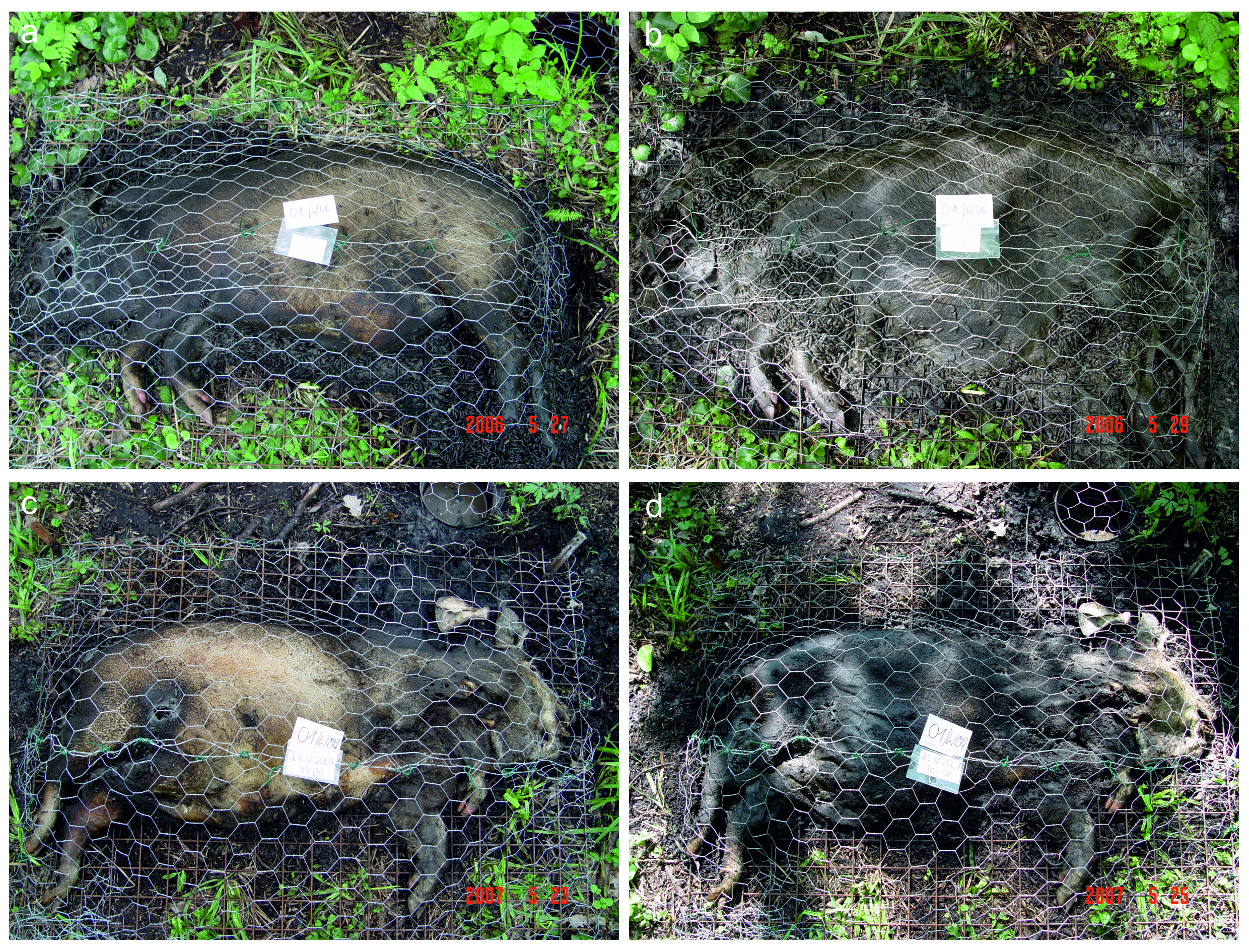


**Fig. II.** Monopolization of pig carcasses by *Necrodes littoralis.* **a,b** – Carcass completely monopolized by larvae of *N. littoralis*, **a** – 34 day of decomposition, larval masses of *N. littoralis* visible near the hind legs, larval exudates cover large parts of the cadaver, **b** – 36 day of decomposition, larval masses visible near the legs (fore and hind) and the neck, exudates cover the entire cadaver. **c,d** - Carcass monopolized in part by larvae of *N. littoralis*, the anterior part of the cadaver was consumed by larval blow fies, **c** – 38 day of decomposition, exudates of larval *N. littoralis* cover parts of the trunk and hind legs, **d** – 40 day of decomposition, larval exudates cover the entire trunk and hind legs.


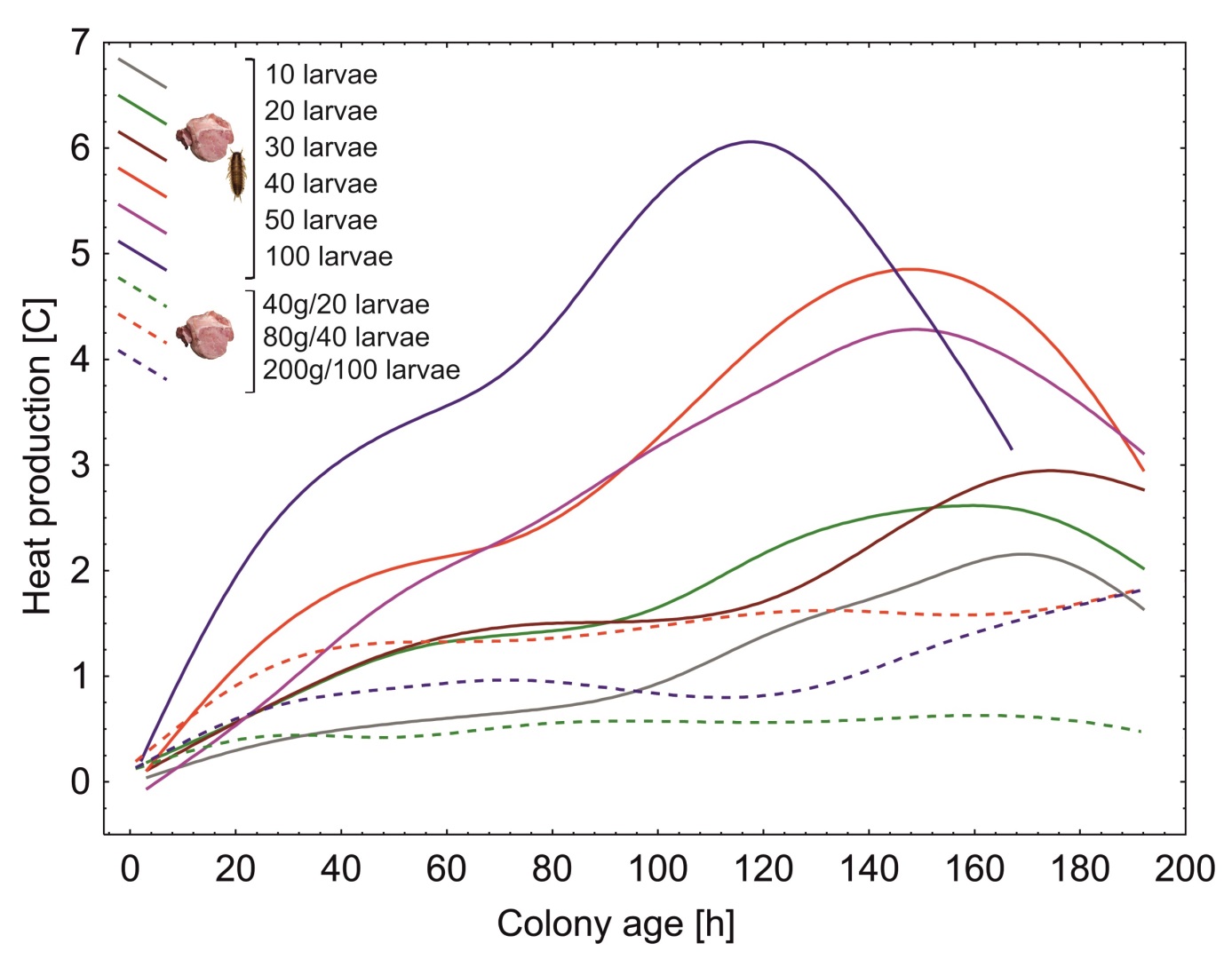


**Fig. III.** Longitudinal thermal profiles of the feeding matrix in colonies of larval *Necrodes littoralis* with different numbers of larvae (solid lines) and control colonies with meat only (dashed lines). Colonies were reared at room temperature (20-23°C). Thermal profiles were fitted to the data using the distance-weighted least-squares smoothing procedure.
